# Research on the dynamic characteristics of a wind turbine drivetrain based on different control strategies for the generator-side inverter

**DOI:** 10.1371/journal.pone.0347737

**Published:** 2026-05-11

**Authors:** Shijie Zhang, Shuangshuang Jia, Yuhao Zhao, Jing Wei, Yuting Ma, Lan Wu

**Affiliations:** 1 School of Mechanical and Electrical Engineering, Henan University of Technology, Zhengzhou, China; 2 School of Information Technology, Luoyang Normal University, Luoyang, China; 3 College of Mechanical and Vehicle Engineering, Chongqing University, Chongqing, China; CINVESTAV IPN: Centro de Investigacion y de Estudios Avanzados del Instituto Politecnico Nacional, MEXICO

## Abstract

Within the operational context of wind turbines (WTs), the dynamic properties of the drivetrain significantly influence the overall system stability. The implementation of both active and passive control mechanisms within a WT facilitates smooth drivetrain operation. Various methods exist for controlling WTs to ensure optimal performance. This study is focused on the drivetrain of an 8 MW WT, and vibration control is examined as the primary research objective. A dynamic model of the electromechanical system associated with the WT drivetrain is developed. This research focuses on controlling the generator inverter to reduce the difference in immediate torque between the mechanical output torque and the electromagnetic torque (ET) of a permanent magnet synchronous generator (PMSG). Additionally, this study aims to decrease the speed variation between the generator rotor and the output shaft of the gear system to enhance tracking precision. A resonant integrator is designed to introduce specific harmonics to mitigate the harmonics present in both the ET and the current of the generator, ultimately enhancing efficiency. The results of this research demonstrate that the proposed control strategy effectively reduces transverse vibrations in critical components of the gear system while suppressing harmonics in generator current and electromagnetic fields. This approach is a viable solution for the active reduction in vibrations within the WT drivetrain.

## 1. Introduction

With the occurrence of continuous energy crises, escalating environmental pollution, and increasing climate change issues, countries worldwide are actively exploring various methods and for addressing energy, environmental, and climate challenges. Among the various strategies identified, the mitigation of fossil fuel combustion and the advancement of the development and utilization of clean energy sources have been recognized as essential [1]. In contrast to alternative clean energy sources, wind energy is highly sought after by nations worldwide because of its minimal geographical limitations, substantial resource availability, and relatively straight forward technological development process. Furthermore, wind energy has the fastest growth rate among the forms of clean energy. With the progressive maturation of wind power technology, to reduce unit cost, increase the land utilization rate, and obtain additional wind energy, the development of single-unit high-power turbines (WTs) has increased [2–3]. The large scale of WTs indicates that they are certain to move toward the development direction of high efficiency, high reliability, and high precision. In addition, WTs will exhibit a lightweight design [4]. The combination of high power and low weight is likely to exacerbate the coupled vibrations within the WT drivetrain components. Such pronounced vibrations may result in varying levels of damage to the WT system. Engineering practice suggests that fully understanding the equipment under the actual working conditions of force, vibration response, and other dynamic behavior is key to equipment optimization [5]. Dynamic behavior abnormalities and dynamic instability are important factors that cause the shutdown maintenance of WTs and harm the safety of the entire machine. Dynamic stability analysis, vibration control technology research, dynamic design, and optimization of the mechanical and electrical parameters of WTs have become popular issues in industry and academia [6].

Before examining the dynamic characteristics of WTs, it is crucial to establish an accurate dynamic model to support thorough research. Qin et al. formulated a model of the WT drivetrain that incorporates the elasticity of gears and bearings to examined the influences of intrinsic characteristics, modal shapes, and internal dynamic excitations on the transmission system. However, the models they proposed did not include the generator, and they failed to consider the interactions between electrical and mechanical systems, which led to certain limitations in their findings [7].. Before examining the dynamic characteristics of WTs, it is crucial to establish an accurate dynamic model to support thorough research. Rahimi and Parniani identified the variables leading to system instability and the factors that could cause system motion instability by analyzing the dynamic behavior of fixed-speed WTs [8]. Helsen et al. analyzed the impacts of the flexibility levels of different components within the WT on the transmission system. Subsequently, they developed a test bench for the WT gearbox, to analyze the interplay between mechanical dynamics and electrical control within the transmission chain of the WT [9–10]. Guerine established a dynamic model for the concentrated mass of an 8-degree-of-freedom WT and introduced an interval analysis approach to assess the dynamics of a WT transmission chain system characterized by uncertain yet constrained parameters [11]. Zhao and Ji established a transmission chain model of a WT with a pure torsion gearbox, and evaluated how both internal and external factors influence the movement of the transmission system. However, Zhao and others considered only a pure torsional model of the gearbox without considering the lateral vibration displacement (VD) of the gearbox or performing coupling generator coupling [12]. Yan et al. proposed a hybrid model that integrates both physical and data-driven approaches to simulate the dynamic operational characteristics of WTs. This methodology effectively captures and learns the time-series dynamic characteristics associated with the operating conditions and parameters of WTs across varying wind conditions. Consequently, this method improves both the accuracy of simulations and the computational efficiency regarding the load dynamic characteristics of WTs [13]. Awalin et al. studied the voltage and current of a dynamic model of WTs. The scholars discussed the influences of different WT rotating speeds on the voltage and current output, established a WT model in PSCAD, and validated the accuracy of the model by comparing the WT active power with manually calculated results [14]. Javier et al. proposed a simplified linear time-domain dynamic model for offshore floating WTs. The model accurately predicts the movement of WTs with wind and waves, with a prediction accuracy error of less than 10% under all the considered loads [15]. These scholars have almost all simplified the electrical system model, resulting in some distortion of the results. Therefore, this research will establish a more detailed electrical system in the model.

With the development of flexible multibody dynamics theory and the rapid expansion of the wind power industry, many scholars have begun to investigate the vibration characteristics of WTs to suppress their vibration and make their operation more stable. In their investigation of vibration sources associated with large variable speed wind turbines (WTs), Xing et al. developed a proportional–integral–differential (PID) controller integrated with a torque damping filter. This design seeks to mitigate the transmission load experienced by 1.5 MW WTs to effectively manage vibrations [16]. Yip et al. introduce a direct torque control methodology that the proposed approach is substantiated through MATLAB numerical simulations and a microscale WT simulator, with demonstrating the efficacy of the model in optimizing wind energy extraction [17]. Jin et al. developed a generator torque controller based on LQR to improve the damping characteristics of the transmission chain, consequently mitigating dynamic loads [18]. Song et al. introduced a new current controller into the double fed WT that could actively compensate for external disturbances on the basis of the turbine operating state, mitigating the impact of grid disturbances on the transmission chain. In the model, the mechanical elements of the WT drivetrain are represented by two mass blocks. This approach does not account for the interactions between the mechanical components [19]. Ruban periyanayagam and Joo proposed a novel integral sliding mode control (SMC) method for optimal wind speed tracking of WTs and for reducing mechanical loads on WTs. The WT model was validated using MATLAB/Simulink [20]. Chatri et al. proposed a high-order SMC method, which is utilized for generator-side converters and grid-side converters to reduce mechanical torque and system chattering problems [21]. Ren et al. utilized the Hamilton model to investigate the vibration suppression of offshore WT towers under wind and wave excitations, confirming the effectiveness of the control strategy [22]. Menezes and Araujo combined H-8 control design with WT dynamic equations to create an active structural control system that reduces tower loads, demonstrating its effectiveness under stable and turbulent conditions. A shortcoming is that they did not consider the influence of the gearbox and generator on the impeller [23]. Wang et al. introduced a dynamic integrated inertia control approach aimed at mitigating frequency fluctuations in low-power systems, and the efficacy of this method was subsequently validated through empirical analysis. This model ignored the interaction between the mechanical parts inside the transmission chain [24]. Queija et al. analyzed and reviewed the latest developments in control technology for floating offshore WTs, examining the benefits and drawbacks of commonly used control methods while forecasting future research trends [25]. Zhang and his associates created a synovial-enhanced model reference adaptive system observer. The efficacy of the proposed control strategy was validated through simulation tests. However, it is crucial to acknowledge that these scholars did not extend the validation of the control strategy to WTs [26]. Xie et al. proposed an innovative WT control methodology that leverages reinforcement learning by incorporating an online-trained deep neural network within a model predictive control framework. However, this method omits the nonlinearities and contact dynamics inherent in the gear transmission system [27]. Ai et al. recommended integrated single-paddle control and pitch control strategies based on an open- source OpenFAST platform. Their approach incorporates generator speed control, which employs an active anti-interference control algorithm for the regulation of generator speed. The results indicated that the proposed control strategy outperformed traditional methods [28]. Chen et al. introduced a sensorless control strategy for variable-frequency synovium utilizing extended state observer compensation. This approach involved the implementation of a rapid exponential arrival law within the velocity loop and the incorporation of an integrated synovium surface. However, the authors did not consider the interactions among the mechanical components within the WT, such as gear meshing forces and impact loads [29]. Tang et al. established an 8-DOF model for WTs and used NREL 5MW WTs as the benchmark model to integrate pitch angle control and a dynamic shock absorber to control WT vibration; the scholars reported that the combination of active pitch control and a tuned mass damper could effectively reduce the structural response and improve generator performance [30]. Wang introduced an innovative control strategy for the machine-side converter, which enhanced the motor’s inertia to mitigate power oscillations. Concurrently, simulation analyses conducted with the DIgSILENT Power Factory corroborated the efficacy of the new control strategy [31]. Gao et al. developed a linearized small-signal model for a dual-fed induction WT utilizing hybrid synchronous control, incorporating both rotor speed regulation and a model of the transmission system. Their analysis was focused on the interplay between the grid strength and transmission system, revealing the presence of two resonance peaks within the system [32]. By developing a fourth-order dynamic model of the nonlinear system associated with WTs, Li et al. proposed a fuzzy synovial controller and an adaptive synovial controller. They researchers validated the feasibility and effectiveness of their proposed methodology through both simulation analysis and experimental research [33]. Unlike previous scholars, those scholars mainly studies the influence of control methods on the vibration of WTs, and many studies ignore the gear transmission system, resulting in the inability to reflect nonlinear phenomena within the transmission system. Therefore, this article will take into account the nonlinear phenomena of the transmission system when modeling.

In summary, in previous studies, most scholars have often only established detailed models of the target object while extensively simplifying non-target objects, even ignoring their intrinsic incentives. As a result, the results obtained have had significant bias or incompleteness. In response to this issue, this paper develops a comprehensive coupling model that integrates the aerodynamic model of the WT impeller, the mechanical transmission chain model, and the electrical system. Unlike previous studies that add dampers for active vibration suppression, this paper does not introduce additional components. Instead, it minimizes the speed and torque differences between the transmission system and the generator by adjusting the inverter on the WT generator side, thereby avoiding increased hardware costs. Furthermore, while most prior research has primarily focused on torsional vibration responses, this study concentrates on the lateral vibration of the system and examines the impact of different control strategies on the lateral vibration of the transmission system. The remainder of this research is organized as follows. Section 1 provides a summary of the present state of development concerning WT drivetrain modeling, vibration control, and generator control. Section 2 details the mathematical theoretical model pertinent to each component of the WT. Section 3 presents the control strategy design of the generator, from which a new dual control strategy combining a variable-structure sliding film and a resonance controller is proposed. Section 4 features a validation of the feasibility of the strategy through simulation experiments. Section 5 presents the conclusions.

## 2. Theoretical model establishment

### 2.1 Pneumatic model

For WTs, the kinetic energy generated by the wind flow directly determines the maximum capture of wind energy by the impeller. Since the kinetic energy absorbed by the impeller is equal to the kinetic energy lost by the wind, the following applies:


$$\Delta E=\frac{\text{1}}{\text{2}}m_{Q}\overset{\text{2}}{\underset{Q}{v}}-\frac{\text{1}}{\text{2}}m_{H}\overset{\text{2}}{\underset{H}{v}}=\frac{\text{1}}{\text{2}}\rho \left(S_{Q}\overset{\text{2}}{\underset{Q}{v}}-S_{H}\overset{\text{2}}{\underset{H}{v}}\right)v$$ (1)

In Equation (1), *m*_*Q*_ is the mass of the air before passing through the impeller, *m*_*H*_ is the mass of the air after passing through the impeller, *S*_*Q*_ is the area before the wind passes through the impeller, *S*_*H*_ is the area after the wind passes through the impeller, *v*_*Q*_ is the velocity of the air before passing through the impeller, *v*_*H*_ is the velocity after passing through the impeller, *ρ* is the density of air, and *v* is the wind speed as it traverses the impeller.

The instantaneous power absorbed by the WTs is as follows:


$$P=\frac{\text{1}}{\text{4}}\rho \left(S_{Q}\overset{\text{2}}{\underset{Q}{v}}-S_{H}\overset{\text{2}}{\underset{H}{v}}\right)\left(v_{Q}+v_{H}\right)=\frac{\text{1}}{\text{4}}\rho \left(S_{Q}\overset{\text{3}}{\underset{Q}{v}}-S_{H}\overset{\text{2}}{\underset{H}{v}}v_{Q}+S_{Q}\overset{\text{2}}{\underset{Q}{v}}v_{H}-S_{H}\overset{\text{3}}{\underset{H}{v}}\right)$$ (2)

After the power function is simplified, the optimal value can be determined.


$$P=\frac{\text{8}}{27}\rho S_{L}\overset{\text{3}}{\underset{Q}{v}}$$ (3)

In Equation (3), *S*_*L*_ is the area swept by the impeller.

The power obtained by scanning the upstream area of the WT is:


$$P_{S}=\frac{\text{1}}{\text{2}}\rho S_{L}\overset{\text{3}}{\underset{q}{v}}$$ (4)

Therefore, the maximum power efficiency of the WT is:


$$\frac{P}{P_{S}}=\frac{\frac{\text{8}}{27}\rho S_{L}\overset{\text{3}}{\underset{q}{v}}}{\frac{\text{1}}{\text{2}}\rho S_{L}\overset{\text{3}}{\underset{q}{v}}}=\frac{16}{27}$$ (5)

The maximum utilization rate of wind energy, also known as the Betz theory, indicates that the kinetic energy obtained by WTs from the wind during operation is limited [34–35].

From equations (4) to (5), it can be seen that the power absorbed by the WT impeller from the wind is:


$$P=\frac{\text{1}}{\text{2}}\rho \pi {R_{y}}^{\text{2}}v^{\text{3}}C_{P}$$ (6)

The torque output from the impeller to the main shaft is as follows [36]:


$$T_{m}=\frac{P}{\omega }=\frac{\frac{\text{1}}{\text{2}}\rho \pi {R_{y}}^{\text{2}}v^{\text{3}}C_{P}}{v\lambda_{f}/R_{y}}=\frac{\frac{\text{1}}{\text{2}}\rho \pi {R_{y}}^{\text{3}}v^{\text{2}}C_{P}}{\lambda_{f}}=\frac{\text{1}}{\text{2}}\rho \pi {R_{y}}^{\text{5}}\omega^{\text{2}}C_{P}{\left(\frac{\text{1}}{\lambda_{f}}\right)}^{\text{3}}$$ (7)

In Equation (7), *C*_*p*_ is the coefficient of wind energy utilization; *Ry* is the radius of the impeller; *λ*_*f*_ is the tip speed ratio; and *ω* is the rotational speed of the impeller.

The utilization rate of wind energy is typically expressed using an empirical formula [37]:


$$\left\{ \begin{array}{c} @l@C_{P}=0.5176\left(\frac{116}{\zeta }-0.4\beta -5\right)e^{\frac{-21}{\zeta }}+0.0068\zeta \\ \frac{\text{1}}{\zeta }=\frac{\text{1}}{\lambda_{f}+0.08\beta }-\frac{0.035}{\beta^{\text{2}}+1}\\ \lambda_{f}=\frac{\omega R_{y}}{v} \end{array}\right.$$ (8)

In Equation (8), *β* is the pitch angle.

### 2.2. Mechanical transmission chain model

**Fig 1:**
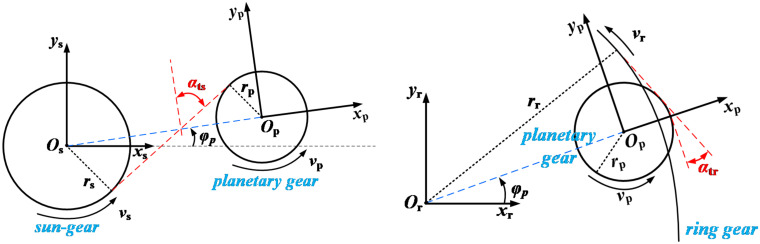
Relative displacement of the toothed components within a PG system. This image illustrates the meshing of gears within the transmission chain system. (a) shows the meshing and rotation of the sun-gear and planetary gears in the planetary gear train. (b) shows the meshing and rotation of the planetary gears and the inner gear ring in the planetary gear train.

The impeller is substituted with an equivalent mass and equivalent torque [38]. In the context of the transmission system, the kinematic relationships governing the interactions between the sun gear (SG) and the planetary gear (PG), and between the PG and the ring gear (RG), are shown in Fig 1. The dynamic equation of the planetary gear transmission system is shown in Appendix A.

The mechanical rotor of the generator is influenced by the ET and input torque. Neglecting the frictional force, the differential equation of the generator rotor is as follows:


$$\left\{ \begin{array}{c} @l@m_{gen}{\ddot{x}}_{gen}+c_{gen}{\dot{x}}_{gen}+k_{g12x}x_{gen}=0\\ m_{gen}{\ddot{y}}_{gen}+c_{gen}{\dot{y}}_{gen}+k_{g12y}y_{gen}=-m_{gen}g\\ J_{gen}{\ddot{\theta }}_{gen}+c_{gen}({\dot{\theta }}_{\text{1}}-{\dot{\theta }}_{\text{2}})+k_{gen}(\theta_{\text{1}}-\theta_{\text{2}})=T_{m} \end{array}\right.$$ (9)

In Equation (9), *m*_*gen*_ representsthe rotor mass of the generator; *k*_*gen*_ represents the torsional stiffness of the generator shaft; *c*_*gen*_ denotes the torsional damping of the generator shaft; *θ*_*1*_ and *θ*_*2*_ represent the torsional angular displacements at the front and rear ends of the generator shaft, respectively; *k*_*g12x*_ and *k*_*g12y*_ represent the stiffness of the rotor support in the *X* and *Y* directions, respectively; *J*_*gen*_ represents the moment of inertia of the rotor; and *T*_*m*_ represents the mechanical torque.

### 2.3. Generator and control system model

The high-speed shaft of the mechanical transmission system is linked to the rotor of the generator via coupling, and the mechanical torque is input into the generator to drive its movement and generate electrical energy. With respect to the generator, when the air gap is uniform, the 3-phase static model of the generator can be expressed as follows [39]:


$$\left[\begin{array}{c} @c@u_{a}\\ u_{b}\\ u_{c} \end{array}\right]=\left[\begin{array}{ccc} @ccc@R_{s} & & \\ & R_{s} & \\ & & R_{s} \end{array}\right]\left[\begin{array}{c} @c@i_{a}\\ i_{b}\\ i_{c} \end{array}\right]+\left[\begin{array}{c} @c@\left(L_{s\sigma }+1.5L_{m}\right){\dot{i}}_{a}-\phi_{f}{\dot{\theta }}_{Gr}sin\theta_{Gr}\\ \left(L_{s\sigma }+1.5L_{m}\right){\dot{i}}_{b}-\phi_{f}{\dot{\theta }}_{Gr}sin\left(\theta_{Gr}-120^{\circ }\right)\\ \left(L_{s\sigma }+1.5L_{m}\right){\dot{i}}_{c}-\phi_{f}{\dot{\theta }}_{Gr}sin\left(\theta_{Gr}+120^{\circ }\right) \end{array}\right]$$ (10)

In Equation (10), the variables *u*_*a*_, *u*_*b*_, and *u*_*c*_ denote the 3-phase voltages of the PMSG. The symbol *R*_*s*_ denotes the resistance of the generator rotor, whereas *i*_*a*_, *i*_*b*_, and *i*_*c*_ correspond to the 3-phase currents of the PMSG. The term *ϕ*_*f*_ refers to the stator flux linkage, and *θ*_*Gr*_ indicates the phase difference among the three phases. Additionally, *L*_*sσ*_ represents the leakage inductance associated with the stator winding of the generator, and *L*_*m*_ denotes the equivalent excitation inductance of the generator.

The grid voltage is obtained through phase-locking when the voltage vector is utilized for control. When the phase-locked loop is employed, the ideal state grid voltage phase angle aligns precisely with the phase-locked angle. The grid voltage in the 2-phase rotating coordinate system is as follows:


$$\left\{ \begin{array}{c} @l@e_{gd}=-L_{gd}\frac{di_{gd}}{dt}-R_{g}i_{gd}+E_{g}+\omega_{g}L_{gq}i_{gq}\\ e_{gq}=-L_{gq}\frac{di_{gq}}{dt}-R_{g}i_{gq}-\omega_{g}L_{gd}i_{gd} \end{array}\right.$$ (11)

In Equation (11), *E*_*g*_ is the voltage amplitude.

### 2.4. Electromechanical control coupling model

**Fig 2:**
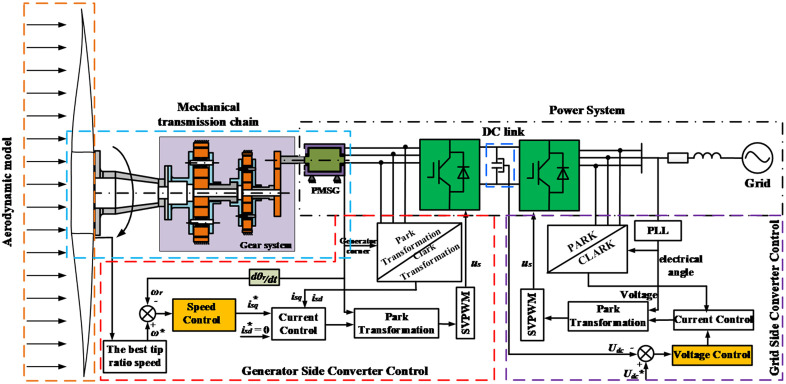
Coupling model of the machine –electricity –control -grid of a WT drivetrain. The diagram presents a schematic of the coupled wind turbine drivetrain system, encompassing the aerodynamic, mechanical, electrical, control, and grid components. From front to back, it illustrates the aerodynamic load model, mechanical transmission system model, generator model, generator converter control, DC decoupling stage, grid-side converter control, and grid model, respectively.

The aerodynamic model, the equivalent impeller model, the main shaft model, the drivetrain model, the generator model, the inverter control model, and the grid model are integrated to form the electromechanical control coupled dynamics model, as shown in Fig 2. The aerodynamic model uses the rated wind speed, and the mathematical model for this part is explained in Section 2.1. The mechanical transmission chain system represents a gear transmission system, and the theoretical model of this system is described in Section 2.2, which separately describes PG systems and parallel gears. The theoretical models of the generator-side converter controlling the generator and the grid-side converter controlling the power grid are described in detail in Section 2.3. Through data transmission and exchange, a WT transmission chain electromechanical coupling dynamic model is ultimately established. In this framework, the disparity in rotational speed between the rotor of the generator and the high-speed shaft of the gearbox can be minimized through the alteration of the generator’s control strategy. This phenomenon results in a more effective rotor tracking effect for the generator.

**Fig 3:**
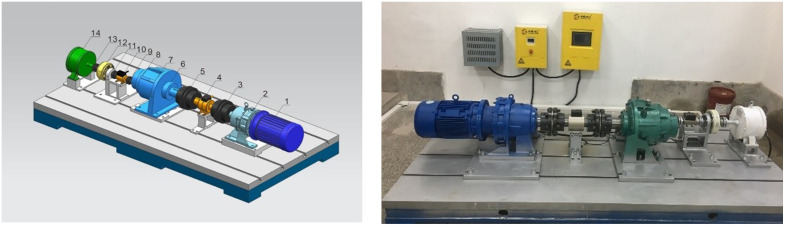
Design of the drivetrain test bench and physical test bench. (1 – Drive motor; 2 -Reducer; 3, 5 – Coupling 1; 4, 10 – Speed and torque sensor; 14 – Generator; 9, 11, 13 -Coupling 2; 12 – Magnetic powder brake; 6, 7, 8 – Gear speed reducer).

The test bench is designed according to the drivetrain diagram of the WT. Due to the limitations of laboratory conditions, it is not possible to simulate the impeller of a WT. Additionally, the mechanisms through which generator control affects the dynamics of the electromechanical system of the WT drivetrain are primarily examined. To facilitate this investigation, an electric motor is employed to replicate the blade torque input of the WT during the assembly of the test platform, as shown in Fig 3. The drivetrain model is confirmed through testing on the bench shown in Fig 3, as explained in the literature [40].

## 3. Design of controllers

After validating the model on the test bench, the model parameters were adjusted to match those of an actual WT, forming an electromechanical coupled model of the 8MW WT. Controller design and control method research were then conducted on the newly developed model. Due to the lack of conditions for conducting industrial-grade testing on an 8MW WT, the WT was ultimately verified through numerical simulation. Although simulation verification alone cannot fully reflect the operational status of real WTs, it can provide guidance for analyzing the operational status of real WTs. The detailed controller design is as follows.

### 3.1. PID controller design

The generator in the WT drivetrain is usually controlled using a PID controller. The expression is as follows:


$$u\left(t\right)=K_{p}\left[e\left(t\right)+\frac{\text{1}}{T_{i}}\int_{\text{0}}^{t}e\left(t\right)dt+T_{d}\frac{de\left(t\right)}{dt}\right]=K_{p}e\left(t\right)+K_{i}\int_{\text{0}}^{t}e\left(t\right)dt+K_{d}\frac{de\left(t\right)}{dt}$$ (12)

In Equation (12), *e(t)* is the difference between the input of the system and its corresponding output. The parameter *K*_*p*_ denotes the proportional coefficient. *K*_*i*_ signifies the integration coefficient, defined as *K*_*i*_ = *K*_*p*_/*T*_*i*_, where *T*_*i*_ is the integration time constant. Additionally, *K*_*d*_ denotes the differential coefficient, expressed as *K*_*d*_ = *K*_*p*_*T*_*d*_, where *T*_*d*_ represents the differential time constant.

In the process of modifying the parameters of the current loop, it is essential to first derive the voltage equation after the decoupling of the *d-q* axis.


$$\left\{ \begin{array}{c} @l@u_{d}=\left(K_{d}+\frac{K_{id}}{s}\right)\left(\overset{*}{\underset{d}{i}}-i_{d}\right)-\omega_{e}L_{q}i_{q}\\ u_{q}=\left(K_{q}+\frac{K_{iq}}{s}\right)\left(\overset{*}{\underset{q}{i}}-i_{q}\right)+\omega_{e}\left(L_{d}i_{d}+\phi_{f}\right) \end{array}\right.$$ (13)

By introducing feedback control variables in current control, the system control equation under a PI controller is as follows:


$$\left\{ \begin{array}{c} @l@u_{d}=R_{s}i_{d}-\omega_{s}L_{q}i_{q}+K_{d}\left(\overset{*}{\underset{d}{i}}-i_{d}\right)+K_{id}\int \left(\overset{*}{\underset{d}{i}}-i_{d}\right)dt\\ u_{q}=R_{s}i_{q}+\omega_{s}L_{d}i_{d}+\omega_{s}\phi_{f}+K_{q}\left(\overset{*}{\underset{q}{i}}-i_{q}\right)+K_{iq}\int \left(\overset{*}{\underset{q}{i}}-i_{q}\right)dt \end{array}\right.$$ (14)

In Equations (13) and (14), *K*_*d*_ and *K*_*q*_ are the proportional coefficients of the controller; *K*_*d*_ = *αL*_*d*_, *K*_*id*_ = *αR*, and *α* is the bandwidth of the generator current loop; *K*_*id*_ and *K*_*iq*_ are the integral coefficients of the controller; *K*_*q*_ = *αL*_*q*_; *K*_*iq*_ = *αR*; and *R* is the resistance of the generator’s stator.

The speed PID controller has been developed utilizing the *id* = 0 vector control methodology, and the motion equation of the PMSG [41] is as follows:


$${\dot{\omega }}_{m}=\frac{3p_{n}\phi_{f}}{2J_{gen}}i_{q}-\frac{B}{J_{gen}}\omega_{m}$$ (15)

$$T_{e}=\frac{3p_{n}i_{q}i_{d}}{2J_{gen}}\left(L_{d}-L_{q}\right)+\frac{3p_{n}i_{q}\phi_{f}}{2J_{gen}}$$ (16)

In Equations (15) and (16), *ω*_*m*_ represents the angular velocity of the generator rotor, *J*_*gen*_ refers to the moment of inertia associated with the mechanical rotor of the generator, and *B* signifies the damping coefficient.

When the generator is unloaded, the motion equation of the PMSG is as follows:


$${\dot{\omega }}_{m}=\frac{3p_{n}\phi_{f}}{2J_{gen}}\left({i^{\prime }}_{q}-B_{a}\omega_{m}\right)-\frac{B}{J_{gen}}\omega_{m}$$ (17)

At this juncture, the transfer function relating the generator speed to the *q*-axis current is as follows:


$$\omega_{m}\left(s\right)=\frac{3p_{n}\phi_{f}}{2J_{gen}(s+\beta )}{i^{\prime }}_{q}\left(s\right)$$ (18)

On the basis of Equations (17) and (18), the formulation for the PI controller within the speed loop can be derived.


$$\overset{*}{\underset{q}{i}}=\left(\frac{2\beta J_{gen}}{3p_{n}\phi_{f}}+\frac{\beta K_{p}}{s}\right)\left(\overset{*}{\underset{m}{\omega }}-\omega_{m}\right)-B_{a}\omega_{m}$$ (19)

**Fig 4:**
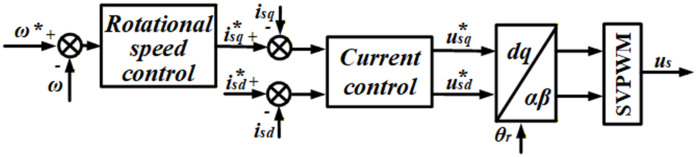
PI control of machine-side converters.

In accordance with the *i*_*d*_ = 0 vector dual closed-loop control, Fig 4 illustrates the control of the inverter on the generator side.

### 3.2. Design of the variable structure SMC

The variable structure SMC is characterized by its straightforward algorithm, rapid response capabilities, and ease of implementation. Additionally, the sliding mode can be used to construct an autonomous dynamic system [42]. Design of a variable structure SMC using a PMSG model in 2-phase rotating coordinates. The mathematical representation of the PMSG in *d*-*q* coordinates is as shown in Appendix C. The sliding surface is defined as follows:


$$s=c_{\text{1}}x_{\text{1}}+x_{\text{2}}$$ (20)

To ensure that the system has good dynamic quality, a variable coefficient exponential convergence relationship is adopted:


$$\dot{s}=-{\left|x\right|}^{a}\varepsilon sgn\left(s\right)-{\left|x\right|}^{b}ks$$ (21)

The speed loop sliding mode control law can be obtained from (20) to (21) as follows:


$${\dot{i}}_{q}=\frac{2J}{3p_{n}\phi_{f}}(-{\left|x_{\text{1}}\right|}^{a}\varepsilon sgn(s)-{\left|x_{\text{1}}\right|}^{b}ks-c_{\text{1}}x_{\text{2}})$$ (22)

According to stability theory, to ensure system stability, it is necessary to satisfy the following:


$$\dot{V}=s\dot{s}=s\left(c_{\text{1}}x_{\text{2}}+1.5p_{n}\phi_{f}/J{\dot{i}}_{q}\right)=-s\left({\left|x_{\text{1}}\right|}^{a}\varepsilon sgn\left(s\right)+{\left|x_{\text{1}}\right|}^{b}ks\right)$$ (23)

Therefore, as long as *ε* > 0 and *k* > 0, the condition can be satisfied, and equation (22) becomes the following:


$$i_{q}=\frac{2J}{3p_{n}\phi_{f}}\int (-{\left|x_{\text{1}}\right|}^{a}\varepsilon sgn(s)-{\left|x_{\text{1}}\right|}^{b}ks-c_{\text{1}}x_{\text{2}})$$ (24)

**Fig 5:**
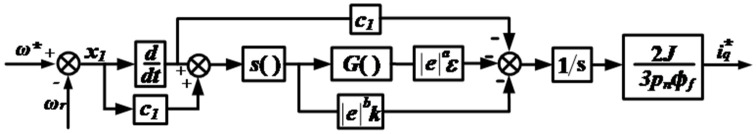
Structural diagram of the exponential SMC with variable coefficients.


To address the chattering phenomenon that is associated with SMC techniques, the sign function is substituted with the function denoted as (25) (Fig 5).

$$G\left(s\right)=\frac{s}{\left|s\right|+v}$$ (25)

The final variable coefficient variable structure SMC controller current is:


$$i_{q}=\frac{2J}{3p_{n}\phi_{f}}\int (-\frac{{\left|x_{\text{1}}\right|}^{a}\varepsilon s}{\left|s\right|+v}-{\left|x_{\text{1}}\right|}^{b}ks-c_{\text{1}}x_{\text{2}})$$ (26)

In Equations (26), *a* and *b* represent transformation coefficients, and *ε* is the convergence rate coefficient.

### 3.3 Resonant integral controller design

**Fig 6:**
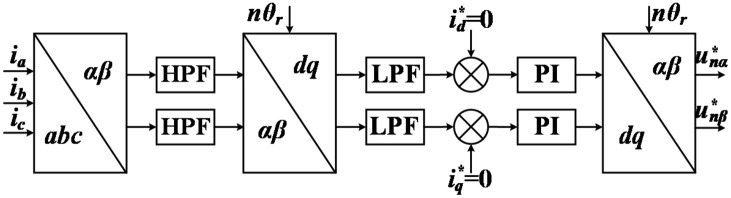
PI control block diagram of the harmonic current.


In a synchronous rotating coordinate system, the proportional-integral (PI) controller is capable of performing effective nondifferential control. The block diagram of the harmonic suppression is shown in Fig 6. This method involves first converting the harmonic current into direct current (DC) signals and then converting the DC signal into an AC signal after being controlled by the controller. For the harmonic conversion control process of the generator current, the transfer function is as follows:

$$G_{ac}\left(s\right)=G_{dc}(s-j\omega )+G_{dc}(s+j\omega )$$ (27)

By converting the integral controller and the nonideal controller through the above formula, the transfer function is obtained as follows:


$$G_{ac}\left(s\right)=\frac{2K_{i}\left(\omega_{c}s+\overset{\text{2}}{\underset{c}{\omega }}\right)}{s^{\text{2}}+2\omega_{c}s+\overset{\text{2}}{\underset{c}{\omega }}+\omega^{\text{2}}}\approx \frac{2K_{i}\omega_{c}s}{s^{\text{2}}+2\omega_{c}s+\omega^{\text{2}}}$$ (28)

Equation (28) represents a nonideal resonant controller. Adjusting the cutoff frequency of this nonideal controller can modify the bandwidth of the controller, decrease the sensitivity of the controller to signal frequency variations, and enhance the overall stability of the system. A nonideal controller and a PI controller can be combined to obtain the proportional integral resonant controller in rotating coordinates, and the transfer function is as follows:


$$G_{ac}\left(s\right)=K_{p}+\frac{K_{i}}{s}+\frac{2K_{i}\omega_{c}s}{s^{\text{2}}+2\omega_{c}s+\omega^{\text{2}}}$$ (29)

By adjusting the nonideal controller, multiple harmonics can be regulated, and the transfer function becomes the following:


$$G_{ac}\left(s\right)=K_{p}+\frac{K_{i}}{s}+\sum_{h=1}^{\infty }\frac{2K_{hi}\omega_{c}s}{s^{\text{2}}+2\omega_{c}s+{\left(h\omega \right)}^{\text{2}}}$$ (30)

In Equation (30), Kp and Ki represent the coefficients for proportional and integral control, respectively, and *K*_*hi*_ is the *h*^*th*^ harmonic vibration coefficient.

### 3.4. New combination control strategy

**Fig 7:**
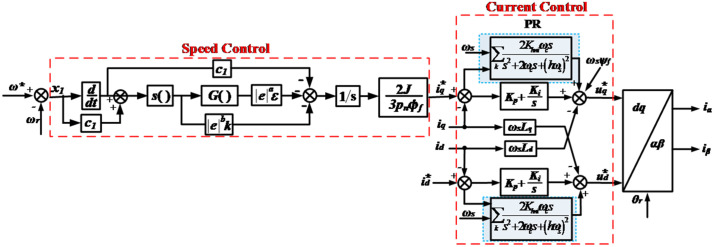
Harmonic control strategy based on the SMC controller. The diagram illustrates an SMC-PR controller, which primarily consists of a speed loop and a current loop. The speed loop is regulated using the SMC method, whereas the current loop is managed using the PR control method.

The variable structure SMC is utilized to control the speed of the generator, thereby reducing vibrations within the drive train. Harmonic suppression is carried out by the current loop, and electrical harmonics in the generator are directionally suppressed. Ultimately, the rotational speed ring and current ring collaborate to fulfill the dual objective of vibration suppression and harmonic suppression of the WT system. Consequently, this process improves the dynamic properties of the WT drivetrain, as shown in Fig 7.

### 3.5. Multi controller parameter tuning and comparison benchmark

To ensure the fairness and scientific rigor of the comparison among the PI, PR, SMC, and SMC-PR control strategies presented in this paper, this section details the principles of parameter tuning. The parameters of the PI and PR controllers are initially designed based on frequency domain theory: the PI controller focuses on the stability margin of the fundamental component, while the PR controller compensates for gain at specific resonant frequencies. The SMC controller determines switching gains based on the upper bound of uncertainty to guarantee the existence of sliding mode motion. For the SMC-PR composite controller, the sliding mode component provides global robustness, whereas the PR component enhances tracking accuracy for specific modes. All controllers aim to minimize the absolute integral error and improve the suppression of disturbances at specific frequencies as a unified optimization objective, performing parameter optimization under identical wind excitation conditions. This approach ensures that all four controllers achieve equivalent optimization levels of optimization within their respective structural frameworks, allowing subsequent performance comparisons to objectively reflect the fundamental impact of differences in control structures.

## 4. Discussion

**Fig 8:**
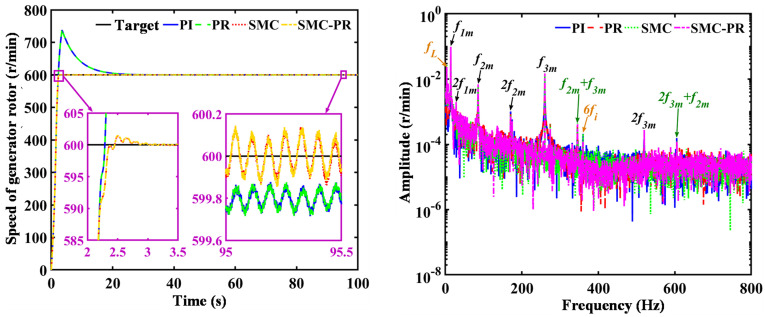
Dynamic behavior of generator rotor speed in response to various control strategies. The figure shows the time-domain and frequency-domain responses of the generator rotor speed under four different control modes.

To enhance research, the effects of various control strategies on the electromechanical dynamic characteristics of a drivetrain are examined exclusively under rated operating conditions. To validate the accuracy of the model, MATLAB/Simulink is employed to develop a theoretical control model for a large WT drivetrain, followed by simulation testing to confirm its reliability. The time-domain analysis of the generator rotor speed, conducted under various control strategies, indicates that the rotor speed remains stable when controlled by both the PI and the proportional resonant (PR) controllers. The parameters of different control strategies are shown in Table 1. Similarly, the generator speed remains stable when SMC and SMC-PR controller control are used. As shown in Fig 8(a), the application of PI control and PR control results in the WT system achieving a stable state approximately 29 seconds after initiation, with the generator speed fluctuating around the desired target value. In contrast, when the system is regulated by SMC and SMC-PR control methods, the WT system reaches stability in approximately 3 seconds, thereby decreasing the time required to reach a stable state by 89.67%, as shown in Table 1. Evidently, that PR control does not impact the generator rotor speed. The frequency domain diagram reveals that under the control of the four strategies, the frequency domain components of the generator rotor speed are essentially the same. The frequency domain primarily encompasses the initial stage PG meshing frequency (*f*_*1m*_) along with its harmonics (2*f*_*1m*_), the second-stage PG meshing frequency (*f*_*2m*_) and its harmonics (2*f*_*2m*_), the PS meshing frequency (*f*_*3m*_) and its harmonics (2*f*_*3m*_), the generator shaft rotation frequency (*f*_*L*_), the harmonics of the generator current (6*f*_*i*_), and the combined frequencies (*f*_*2m*_ + *f*_*3m*_) and (2*f*_*2m*_ + *f*_*3m*_), as shown illustrated in Fig 8(b).

**Table 1:** Parameters of different control strategies.

	Speed loop					Current loop							
PI	P			I		P				I			
	1000			199		80				0.75			
PR	P			I		*K*_*h1*_	*h*_*1*_	*K*_*h2*_	*h*_*2*_	*K*_*h3*_	*h*_*3*_	*K*_*h4*_	*h*_*4*_
	1000			199		1.15	5	195	7	5.5	9	23.15	11
SMC	*a*	*b*	*k*	*ε*	*c*_1_	P				I			
	0.01	1.72	12	0.8	5	80				0.75			
SMC-PR	*a*	*b*	*k*	*ε*	*c*_1_	*K*_*h1*_	*h*_*1*_	*K*_*h2*_	*h*_*2*_	*K*_*h3*_	*h*_*3*_	*K*_*h4*_	*h*_*4*_
	0.01	1.72	12	0.8	5	1.15	5	195	7	5.5	9	23.15	11

**Fig 9:**
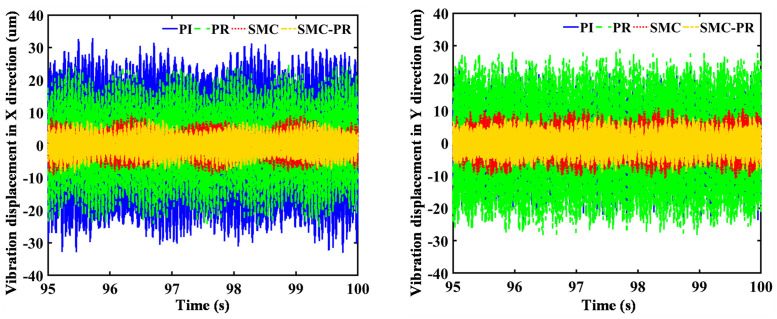
Lateral VD of the first-stage planetary output gear. The figure illustrates the lateral vibration displacement of the first-stage sun gear under four different control strategies employed by the generator.

**Fig 10:**
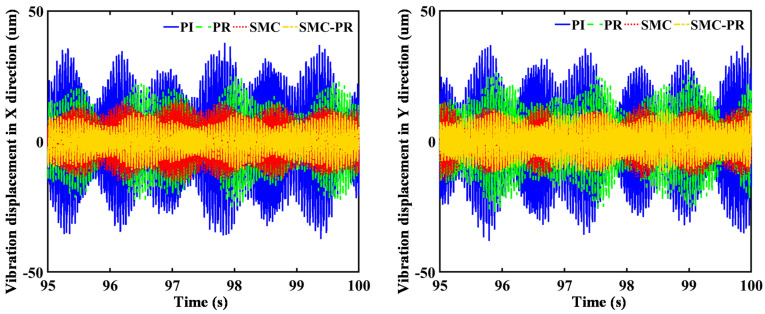
Lateral VD of the second-stage planetary output gear. The figure illustrates the lateral vibration displacement of the second-stage sun gear when the generator adopts four different control strategies.

**Fig 11:**
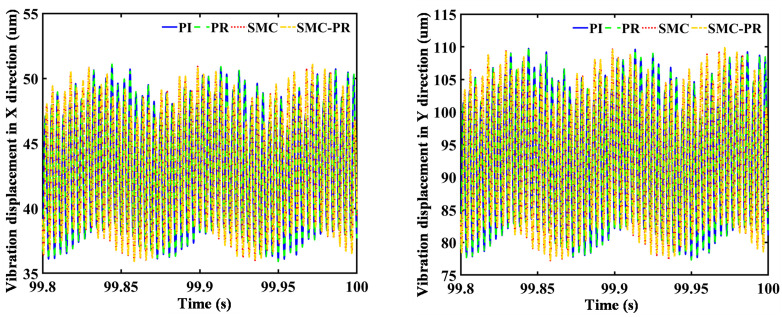
Lateral VD of the PS high-speed gear. The figure shows the lateral vibration displacement of the parallel high-speed wheel gear when the generator employs four different control strategies.

The control strategy does not influence the frequency domain of the rotor speed once the generator reaches a stable state. Therefore, it is unnecessary to consider the speed changes caused by the control strategy when the strategy for harmonic suppression is analyzed. In comparison to the WT utilizing PI control, the implementation of a PR controller results in a 23.77% reduction in the VD amplitude of the first-stage SG along the *X* direction. Conversely, there is an observed increase of 15.50% in the VD in the *Y* direction. The amplitude of the lateral VD for the second-stage SG decreases by 36.24% in the *X* direction and 29.69% in the *Y* direction, as shown in Figs 9 and 10. Similarly, in contrast to a WT using SMC controller control, when the system utilizes SMC-PR control, the lateral VD amplitude of the output gear of the first-stage PG train in the *X* and *Y* directions is reduced by 14.88% and 23.11%, respectively. The lateral VD amplitudes of the output gear of the second-stage PG train in the *X* and *Y* directions decrease by 34.03% and 13.73%, respectively. A comparison of the lateral VD of the output wheel of the PG train when the inverter is controlled by PI and SMC-PR control reveals that when the inverter adopts SMC-PR control, the lateral VD amplitude of the first-stage planetary output gear in the *X* and *Y* directions is reduced by 73.26% and 63.40%, respectively. The lateral VD amplitudes of the second-stage planetary output gear in the *X* and *Y* directions decrease by 70.81% and 64.93%, respectively. The lateral VD of the high-speed gear is not influenced by the change in control strategy (Fig 11). The time required for WTs to reach a stable state under different control strategies is shown in Table 2.

**Table 2:** Reaching steady-state time and maximum overshoot.

	Stability time (s)	Maximum overshoot (r/min)	1-Time(X)/Time(PI) %	1-Overshoot (X)/ Overshoot (PI) %
PI	30.50	139.85	0	0
PR	30.50	139.85	0	0
SMC	3.15	1.85	89.67	98.68
SMC-PR	3.15	1.85	89.67	98.68

**Fig 12:**
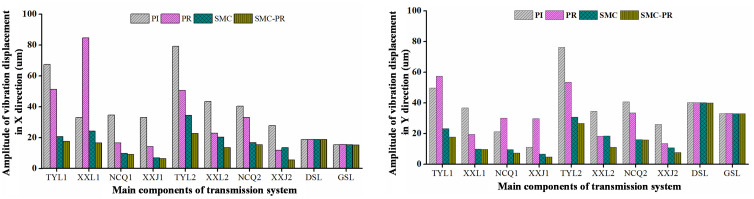
Vibration amplitudes of the primary components within the transmission system.

Under SMC-PR control, the VD amplitude of the toothed components of the PG in both the *X* direction and the *Y* direction is minimized. In comparison, the four control methods applied to the PS gear have a negligible influence on the VD of both the low-speed and high-speed gears in the *X* and *Y* directions. This effect is small enough to be considered inconsequential, as demonstrated in Fig 12. The vibration displacement of each component of the gearbox under different control strategies is shown in Table 3. Furthermore, the PI and PR control methods have distinct effects on the lateral VD of the individual components within the PG system. Specifically, under PR control, there is an observed increase in the VD of the first-stage PG along the *X*-axis, whereas the first-stage SG, RG, and planet carrier increase in the VD along the *Y*-axis. An analysis of the lateral VD of the toothed components in both the first-stage and second-stage PG trains, evaluated under four distinct control methodologies indicates that the VD of the toothed components in the second-stage PG train progressively decreases when subjected to the PI, PR, SMC, and SMC-PR control strategies. Nonetheless, the vibrational displacement of the toothed components within the first-stage PG train remains unaffected by any of the implemented control strategies. This phenomenon can be attributed to the proximity of the first-stage PG train to the impeller of the WT, which strongly affects its performance.

**Table 3:** Vibration amplitudes of the primary components (um).

Control method Parts of Gearbox	PI		PR		SMC		SMC-PR		1-(SMC-PR) / PI %	
	X	Y	X	Y	X	Y	X	Y	X	Y
TYL1	67.87	48.91	51.74	56.49	21.32	23.28	18.15	17.90	73.26	63.40
XXL1	33.64	36.78	84.87	19.52	24.16	10.02	17.22	9.97	48.81	72.89
NCQ1	34.85	21.95	16.93	30.01	10.04	9.86	9.77	7.58	71.97	65.47
XXJ1	33.74	11.90	14.58	29.94	7.24	7.21	7.08	4.96	79.02	58.32
TYL2	79.23	76.68	50.52	53.91	35.06	31.17	23.13	26.89	70.81	64.93
XXL2	43.76	34.25	23.28	17.68	20.12	17.72	13.63	12.11	68.85	64.64
NCQ2	40.65	40.87	33.82	33.67	16.91	17.86	15.25	17.92	62.48	56.15
XXJ2	27.93	26.65	12.38	13.89	13.73	11.12	5.66	7.62	79.74	71.41
DSL	18.51	40.08	18.52	40.02	18.51	40.06	18.51	39.98	0	0.25
GSL	15.23	33.82	15.31	33.86	15.18	33.83	15.08	33.82	0.98	0

**Fig 13:**
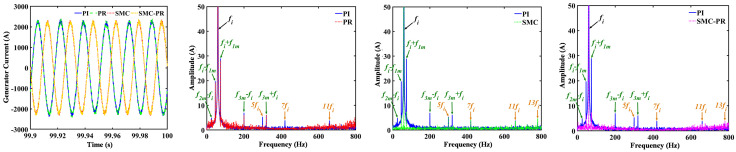
Dynamic responses of the generator current under different control strategies.

The vibration suppression and harmonic suppression methods of the WT drivetrain do not significantly change the time-domain amplitude of the generator current, as shown in Fig 13a. However, compared with the current frequency domain when the generator adopts PI controller control, the harmonics 5*f*_*i*_, 7*f*_*i*_, and 11*f*_*i*_ in the generator current frequency domain when the generator adopts PR control are significantly reduced; in particular, the current harmonics of 5*f*_*i*_ and 7*f*_*i*_ are effectively suppressed. When the generator is regulated using SMC, the combined frequencies *f*_*i*_ ∓ *f*_*1m*_, *f*_*i*_ ∓ *f*_*2m*_, and *f*_*i*_ ∓ *f*_*3m*_, which correspond to the current fundamental frequency and the meshing frequency of the WT drivetrain, are effectively mitigated, resulting in a reduction in the amplitude of these combined frequencies by more than 80%. Furthermore, the implementation of SMC-PR control leads to the suppression of the combined frequency associated with the current fundamental frequency and the meshing frequency of the drive system, while concurrently diminishing the current harmonics at 5*f*_*i*_ and 7*f*_*i*_, as illustrated in Figs 13 (b), (c), and (d).

**Fig 14:**
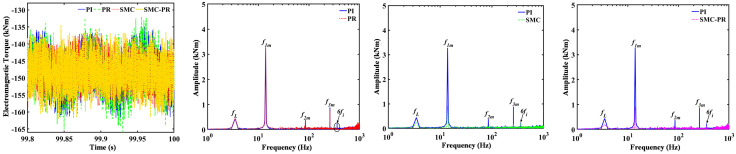
Dynamic responses of the generator ET under different control strategies.

The ET amplitude of the generator exhibits minimal variation in the time domain when different control strategies are implemented, as shown in Fig 14(a). When the generator is regulated using a PI controller, the main components in the frequency domain of the ET include the gear system frequencies *f*_*1m*_, *f*_*2m*_, and *f*_*3m*_, the PS low-speed gear rotation frequency *f*_*L*_ and the torque harmonic 6*f*_*i*_, among which the *f*_*1m*_ amplitude is the greatest. Compared with the frequency domain of the generator ET under PI controller control, the frequency amplitude of the torque harmonic 6*f*_*i*_ in the generator ET under PR control is reduced. When the generator is regulated using SMC, both the meshing frequency and the low-speed rotation frequency of the PS within the ET notably reduce, albeit to varying extents. However, the amplitude of the torque harmonic at 6*f*_*i*_ remains relatively unchanged. In contrast, when the generator employs SMC-PR control, it not only mitigates the meshing frequency present in the ET of the generator but also significantly reduces the harmonic components associated with the ET of the generator.

## 5. Conclusions

The mitigation of vibrations in WT drivetrain systems represents a significant area of inquiry in the field of dynamics research. This study aims to examine the mitigation of vibrations in WT drivetrains through the implementation of active control mechanisms. Moreover, generator harmonic suppression is carried out for the WT, and finally a control strategy that can realize the vibration control of the WT and electrical harmonic suppression is formed.

The vibrational response of WT drivetrains employing various control strategies is compared and analyzed. The simulation findings indicate that when the speed loop is regulated using SMC, the generator exhibits a minimal overshoot in speed and achieves stability more rapidly. Specifically, the time required to achieve stability is reduced by more than 90% in comparison to the implementation of PI control. Moreover, SMC control can effectively suppress the lateral vibration of the planetary output gear in the transmission chain and reduce the lateral VD of the planetary output gear by more than 50%. The current loop using PR control can effectively reduce the current harmonic amplitude in the generator, especially the 5^th^ and 7^th^ harmonic amplitudes of the current, and reduce the 6^th^ harmonic amplitude in the ET, but it cannot suppress the mechanical harmonic in the generator.

The speed loop SMC control and current loop PR control are combined to control the converter. An examination of the vibration response of the WT drivetrain, alongside the dynamic response of the generator, indicates that the SMC-PR methodology is capable of significantly mitigating the lateral vibrations of the output gear within the PG train, achieving a reduction in amplitude exceeding 55%.Moreover, the odd harmonic and combined harmonic *f*_*i*_±*f*_*1m*_ in the generator current are effectively suppressed, and the mechanical harmonic and even harmonic in the ET are suppressed. This finding indicates that the newly developed controller is capable of significantly diminishing the vibrational response of PG components, thereby fulfilling the objective of active vibration mitigation. Moreover, specific harmonics are introduced to directionally suppress the harmonic components of the generator current and ET and reduce the harmonic amplitude of the generator.

## Supporting information

S1 File Appendix A: The dynamic equation of the planetary gear transmission system. Appendix B: The dynamic model of the generator in the 2-phase static model. Appendix C: The mathematical representation of the PMSG in d-q coordinates.
(DOCX)
