# Supplementary material for: Research on the dynamic characteristics of a wind turbine drivetrain based on different control strategies for the generator-side inverter
Source: PLoS One. 2026 May 11;21(5):e0347737. doi: 10.1371/journal.pone.0347737 (PMC13160303; doi:10.1371/journal.pone.0347737)
Supplement: S1 File — Appendix B: The dynamic model of the generator in the 2-phase static model. Appendix C: The mathematical representation of the PMSG in d-q coordinates. (DOCX) [file pone.0347737.s001.docx]

**Appendix A**: The dynamic equation of the planetary gear transmission system

The equations governing the dynamics of the planetary carrier, PG, inner RG, and SG are as follows:

 (A-1)

 (A-2)

 (A-3)

 (A-4)

In Equations (A-1) to (A-4), *m_c_*, *m_p_*, *m_r_*, and *m_s_* represent the masses of the carrier, PGs, RG, and SG respectively; *J_c_*, *J_p_*, *J_r_*, and *J_s_* represent the moments of inertia of the carrier, PGs, RG, and SG, respectively; *x_c_*, *x_p_*, *x_r_*, and *x_s_* denote the radial displacements of the carrier, PGs, RG, and SG in the *X*-direction, respectively; *y_c_*, *y_p_*, *y_r_*, and *y_s_* denote the radial displacements of the carrier, PGs, RG, and SG in the *Y*-direction, respectively; *k_sp_* is the meshing stiffness between the PGs and SG; *k_rp_* is the meshing stiffness between the PGs and RG; *k_xc_* and *k_yc_* denote the support stiffnesses of the carrier; *k_θr_* is the torsional stiffness of the RG; *k_xr_* and *k_yr_* denote the radial support stiffness of the RG; *k_xs_* and *k_ys_* denote the radial support stiffnesses of the SG; *r_c_* represents the base circle radius of the carrier; *F_sp_* is the meshing force between the PGs and SG; *F_rp_* is the meshing force between the PGs and RG; *θ_r_* is the torsional angle of the RG; *α_tr_* is the meshing angle between the PGs and RG; *θ_s_* is the torsional angle of the SG; *α_ts_* is the meshing angle between the SG and PGs; *T_c_* is the input torque to the carrier; and *T_s_* is represent the load torque on the SG.

The motion differential equations for parallel stage (PS) gears are as follows:

 (A-5)

In Equation (A-5), *m_L_* and *m_H_* denote the masses of the gears at the PS; *J_L_* and *J_H_* denote the rotational inertias of the gears at the PS; *k_LH_* represents the meshing stiffness of the gears at the PS; *T_1_* is the input torque of the low-speed gear at the PS; and *T_2_* is the load torque of the high-speed gear at the PS.

**Appendix B**: The dynamic model of the generator in the 2-phase static model

The comprehensive mathematical representation of the PMSG in a 2-phase rotating reference frame can be articulated in the following manner:

 (B-1)

In Equation (B-1), *T_m_* represents the generator input torque, and *B* is the generator friction coefficient. Voltage equation of the grid-side inverter in 2-phase rotating coordinates under symmetric grid voltage conditions:

$\left\{\begin{matrix} e_{gd}=L_{gd}\frac{di_{gd}}{dt}+R_{g}i_{gd}+u_{gd}-\omega_{g}L_{gq}i_{gq} \\ e_{gq}=L_{gq}\frac{di_{gq}}{dt}+R_{g}i_{gq}+u_{gq}+\omega_{g}L_{gd}i_{gd} \\ C\frac{dU_{dc}}{dt}=1.5\left(S_{gd}i_{gd}+S_{gq}i_{gq}\right)-i_{dc} \end{matrix}\right.$ (B-2)

In Equation (B-2), *L_g_* represents the grid-side inductance; *R_g_* denotes the grid resistance; *S_ga_*, *S_gb_* and *S_gc_* are the grid-side inverter switching signals; *e_gd_* and *e_gq_* are the grid voltages on the *d*-axis and *q*-axis, respectively; *i_gd_* and *i_gq_* are the grid currents on the *d*-axis and *q*-axis, respectively; *L_gd_* and *L_gq_* are the inductance components of the grid in 2-phase rotating coordinates; and *ω_g_* is the angular velocity of the grid voltage.

**Appendix C**: The mathematical representation of the PMSG in d-q coordinates.

 (C-1)

When *i_d_*=0, *T_e_*=1.5*p_n_φ_f_*/*i_q_*; when ignoring generator friction, *B_f_* =0. Therefore, for the speed loop control of a PMSG system, the selected generator state variables are as follows:

 (C-2)

There are:

From Equations (C-1) and (C-2), the following conclusions can be drawn:

 (C-3)
